# Exploratory structural equation modeling and the curse of dimensionality

**DOI:** 10.3758/s13428-026-02960-y

**Published:** 2026-03-11

**Authors:** Tra T. Le, Jeroen K. Vermunt, Nicola Ballhausen, Katrijn Van Deun

**Affiliations:** 1 Department of Methodology and Statistics, Tilburg University, Tilburg, The Netherlands; 2 Department of Developmental Psychology, Tilburg University, Tilburg, The Netherlands

**Keywords:** Structural equation modeling, High-dimensional data, Exploratory factor analysis, Regularization

## Abstract

The next-generation approach to research in the behavioral sciences is based on intensive collections of data and complex models characterized by many parameters for a limited sample size. This introduces new challenges for traditional latent-variable methods, as they are found to fail or yield unstable solutions when the number of variables is large relative to the sample size. To tackle this issue, we propose a two-stage regularized approach for exploratory structural equation modeling. In the first stage, we introduce a novel (exploratory) approximate factor analysis technique that not only estimates the measurement model but also the factor scores; indeterminacy of the measurement model is addressed by imposing simple structure through regularizing techniques (LASSO penalty and cardinality constraint). The factor scores can then be used to estimate the structural model in the second stage. An extensive simulation shows that the proposed method outperforms other approaches in recovering the underlying simple structure of the measurement model in both low-dimension high-sample-size and high-dimension low-sample-size settings. The use of the method is demonstrated on two empirical datasets. An implementation of the proposed method in the R software is publicly available: https://github.com/trale97/regularizedESEM.

Research on human behavior and cognition involves studying non-observable constructs such as personality, intelligence, and well-being, which are typically measured through a set of observed indicators. In this setting, structural equation models (SEM) are particularly powerful for building explanatory models, as they model both the latent variables (so-called measurement model) and the relations between the latent variables (so-called structural model). SEM methods are known to work when the number of parameters to estimate is relatively small compared to the sample size. Yet, this condition of “low-dimension high-sample-size” is often not met by modern research designs that make use of technologically advanced measurement tools (e.g., wearable devices tracking one’s physiology and location, genetic sequencing tools, or digital assessment tools used in educational testing) and large data collection (e.g., supplementing questionnaire data with social media data). The resulting data may have more variables than cases, or be so-called high-dimensional, low-sample-size (HDLSS) data. Existing structural equation methods do not work (well) in this setting: the parameter estimates are known to be unreliable when sample sizes are small, and solutions may not even converge (Rosseel, 2020).

The need for SEM for high-dimensional data has been answered in mainly two ways. A first one is based on adaptations of the common factor model, which views the observed variables as reflective indicators of the latent construct. In the common factor model, the loadings are the main parameters of interest and are key to studying the latent variables: Loadings reflect the strength of association between the observed indicators and the unobservable construct. The common approach to obtaining estimates of the loadings requires inverting the observed variables’ (model-implied) covariance matrix, which is an ill-posed problem in the HDLSS setting. Building on the state-of-the-art in computational statistics and machine learning, attempts have been made to adapt such covariance-based SEM methods by adding penalties to the maximum-likelihood (ML) objective functions, such as Regularized SEM (RegSEM Jacobucci et al., 2016), Penalized ML SEM (lslx Huang, 2017; Huang et al., 2020), or Penalized SEM in Mplus (Asparouhov & Muthén, 2024). These approaches, however, often exhibit non-convergence issues when the number of parameters is large relative to the sample size and have high false-positive rates when sample sizes are small (i.e., nonzero loadings are obtained for variables that do not load on the latent construct) (Li & Jacobucci, 2022).

A second common approach to SEM in the setting of HDLSS data is component-based SEM, which represents constructs as weighted sums of observed variables (e.g., poverty index, obesity risk score). Typical methods are partial least-squares SEM (PLS-SEM, Hair et al., 2017), principal component analysis (PCA, Jolliffe et al., 2003), regularized generalized canonical correlation (RGCCA, Tenenhaus et al., 2017), and generalized structural component analysis (GSCA, Hwang & Takane, 2004). In these models, the weights used to form the component scores are the main parameters of interest. An often-named drawback of the component approach is that it is not appropriate for modeling latent variables (Rigdon, 2012; Rönkkö et al., 2015; Sarstedt et al., 2016). Another, not-so-well-known problem is that the component weights are extremely unstable, even with regularization, in the low-dimension, high-sample-size setting. In simulation studies where the composite scores were artificially constructed, zero weights were recovered for variables that were used to form the composite scores, as well as nonzero weights were recovered for variables that did not contribute to the composite scores (Guerra-Urzola et al., 2021; Park et al., 2024). Thus, no meaning can be attached to the size of the weights.

To overcome the aforementioned issues of the currently available SEM methods in the HDLSS settings, we propose a third approach to SEM called Regularized Exploratory Structural Equation Modeling (Regularized ESEM). Two developments in the factor analysis (FA) and SEM literature are key to this proposed approach. First, the estimation of the measurement model is based on the framework of the *approximate factor model* (Chamberlain & Rothschild, 1982; Fan et al., 2021; Bai & Ng, 2023). This is because in the HDLSS setting, factors cannot be estimated with all the properties of the common factor analysis; instead, some assumptions need to be relaxed. Second, inspired by Rosseel and Loh (2022), a two-stage approach is used: the attained factor scores of the approximate factor model are used to estimate the path coefficients in the structural model. The two-stage approach affords applied researchers with flexibility in an exploratory setting, that is, the structural model can be modified (e.g., adding covariates or an outcome) without having to re-estimate the measurement part (Vermunt, 2010). This also helps prevent the meaning of the latent constructs from becoming dependent on the outcome or covariate considered.

The remainder of this paper is structured as follows: first, we will introduce the details of the proposed Regularized ESEM; second, an extensive simulation study will be conducted to evaluate its performance in comparison with other methods; third, the use of Regularized ESEM is demonstrated using two empirical applications, in which one is of an ultra-high-dimensional nature; lastly, the paper ends with a general discussion of the limitations and suggestions for future research. The proposed method was implemented in the R language for statistical computing (R Core Team, 2012). Both the implementation and the code used to generate the results in this paper can be found at https://github.com/trale97/rESEM.

## Method

In this section, we will first introduce the notation and data, followed by the proposed models, their estimation, and model selection.

### Data and notation

The following notations will be used in this paper: matrices and vectors are denoted by bold uppercase and bold lowercase letters, respectively; the transpose is indicated by the superscript $^T$, and scalars by lowercase italics.

The data of interest is denoted by $\textbf{y}_i$, a $J \times 1$ vector containing the scores of person *i* on *J* observed variables. Throughout the paper, we work with standardized data, that is, variables are mean-centered and scaled to unit variance. *S*() is the soft-thresholding operator, which is denoted as $S(x, \lambda ) = \text {sign}(x)(|x|-\lambda )_{+}$ with $\text {sign}(x)$ being the sign of *x* and $(|x| - \lambda )_{+} = \text {max}(0, |x| - \lambda )$.

### Model

Our proposed model is a structural equation model and hence consists of two parts: (1) the measurement model describing the relation between the observed indicator variables and the factors and (2) the structural model describing the relation between the factors (or, with observed variables having no measurement model). First, the measurement model for *Q* factors is expressed as the following model:

1 $$\begin{aligned} \textbf{y} = \textbf{P}\boldsymbol{\eta } + \boldsymbol{\epsilon } \end{aligned}$$

where $\textbf{P}$ is the $J \times Q$ loading matrix, $\boldsymbol{\eta }$ is the $Q \times 1$ vector of factor scores and $\boldsymbol{\epsilon }$ is the $J \times 1$ vector of residuals. Here, no intercept is included because we assume standardized data. Model (1) is subject to two identification constraints: (1) $\textbf{P}$ is assumed to have simple structure, that is, not all observed variables load on all factors but some/many have zero loadings; (2) the factor scores are subject to a length restriction. Furthermore, $\boldsymbol{\epsilon }$ is assumed to have a mean of zero and is uncorrelated with the factor scores. If we write Cov($\boldsymbol{\epsilon })$ = $\boldsymbol{\Phi }$, then we have the model-implied covariance matrix $\boldsymbol{\Sigma } = \textbf{P}\boldsymbol{\Sigma }_{\boldsymbol{\eta }}\textbf{P}^T + \boldsymbol{\Phi }$. Note it is not possible to estimate $\boldsymbol{\Phi }$ as a full rank diagonal matrix in the high-dimensional setting (as in the classical case of a common factor model). Thus, we relax this assumption to allow for $\boldsymbol{\epsilon }$ to be weakly correlated. This type of model is known as the approximate factor model in the econometric literature (Bai & Ng, 2023; Chamberlain & Rothschild, 1982; Fan et al., 2021). Although it differs from the common factor model, the approximate factor model has been shown to produce consistent estimates of loadings and factors under (mild) assumptions in the HDLSS setting (Bai, 2003; Fan et al., 2021).

The structural model expresses the relations among the factors:

2 $$\begin{aligned} \boldsymbol{\eta } = \textbf{B}\boldsymbol{\eta } + \boldsymbol{\zeta } \end{aligned}$$

with $\textbf{B}$ the $Q \times Q$ matrix of path coefficients among the factors and $\boldsymbol{\zeta }$ is an $Q \times 1$ vector of residuals. Note that here to keep the notation simple, Eq. (2) also applies when the structural model includes observed variables instead of factors.

### Stage 1: Estimating the measurement model

The approximate factor model in (1) can be estimated in several ways (e.g., maximum likelihood-based estimation (Bai & Li, 2016), covariance matrix estimation (Fan et al., 2011)). However, for our proposed method, we rely on a least-squares approach as this has been advocated in the high-dimensional and large-scale settings (see Fan et al. (2021)). Hence, we minimize the following objective function:

3 $$\begin{aligned}&\sum _{j = 1}^{J}\sum _{i = 1}^{N}\left( y_{ij}-\sum _q\eta _{iq}p_{jq}\right) ^2\nonumber \\ \text {subject to }&\sum _i\eta _{iq}^2 = N \quad \forall q=1,...,Q, \nonumber \\&\text {and } \textbf{P} \text { showing simple structure}, \end{aligned}$$

with $p_{jq}$ representing the loading of variable *j* and $\eta _{iq}$ the score of person *i* on factor *q*. The loadings are subject to simple structure; some or even many are equal to zero. Apart from sign and permutation invariance, the norm constraint on the factor scores together with the simple structure of the loadings introduces uniqueness^1^. Furthermore, a simple structure facilitates interpretation, especially in settings with an (ultra-) large number of observed variables.

There are several options to formulate a simple structure as a mathematical objective. A direct way to do this is to impose a hard constraint on the number of nonzero loadings; this is looking for a solution with at most *K* nonzero loadings, also known as a cardinality constraint or the best subset problem (Bertsimas et al., 2016a). The best subset problem is generally considered an NP-hard problem and thus is usually approximated by (convex) relaxations. One such popular relaxation is to add the LASSO penalty (Tibshirani, 1996) to the objective function:

4 $$\begin{aligned} \min&\sum _{j=1}^J\sum _{i=1}^N\left( y_{ij}-\sum _q\eta _{iq} p_{jq}\right) ^2 + \lambda \sum _{j=1}^J\sum _{q=1}^Q|p_{jq}| \nonumber \\ \text {subject to }&\sum \eta _{iq}^2=N \quad \forall q=1,...Q, \end{aligned}$$

with $\lambda \ge 0$, the tuning parameter of the LASSO. When $\lambda > 0$, the penalty actively shrinks the loadings toward zero, and some elements to exactly zero. For high values of $\lambda$, (almost) all loadings will become zero. Note that it is not easy to know beforehand how many elements will be zero for a particular value of $\lambda$.

In what follows here, we assume the number of factors *Q* and the value of the LASSO tuning parameter $\lambda$ are given. A strategy to determine these hyperparameters is discussed in Section “Model selection”. The optimization problem defined in (4) is a least-squares low-rank factorization problem with a convex penalty, which can be efficiently solved by alternating optimization (AO, Huang et al., 2016). After initialization, the factor scores and loadings are updated in turn assuming fixed values of the other. To update the factor scores subject to the norm constraint, the method of Lagrange multipliers is used. For the update of the loadings, a coordinate descent procedure (Friedman et al., 2010) is followed. Appendix A details the derivation of the conditional updates of factor scores and loadings, along with the pseudocode of the whole AO procedure. Note that this AO procedure results in a non-increasing sequence of loss values defined in (4) and converges to a stationary point. However, as the exploratory approximate factor analysis problem introduced in (4) is not convex, convergence to the global optimum is not guaranteed and we recommend using multiple starting values to initialize the alternating procedure and retain the solution with the lowest loss value as the final solution.

Orthogonal factors are a special case of model (1), and although our algorithm also applies to this case, we propose to use the algorithm presented by Adachi and Trendafilov (2016) (see also Shen and Huang (2008)). The reason is that this is a particularly elegant method that -exceptionally- allows solving the cardinality-constrained problem with very low computational cost. Furthermore, the use of a cardinality constraint gives users easy control over the number of nonzero loadings and avoids the shrinkage-to-zero bias of the LASSO approach (Bertsimas et al., 2016b). Hence, in the case of orthogonal factors, we solve the cardinality-constrained problem:

5 $$\begin{aligned} {\begin{matrix} \min & \sum _{j=1}^J\sum _{i=1}^N\left( y_{ij}-\sum _q\eta _{iq} p_{jq}\right) ^2\\ \text {subject to } & \sum _i\eta _{iq}^2=N \quad \forall q=1,...Q,\\  & \sum _i\eta _{iq}\eta _{it} = 0 \quad \forall q \ne t, \text { and Card}(\textbf{P})=C, \end{matrix}} \end{aligned}$$

where Card($\textbf{P}$) denotes the number of nonzero coefficients in the loading matrix $\textbf{P}$. Note that here, the factor scores are constrained to be uncorrelated and have unit variance. This implies that when the observed variables are standardized, the nonzero loadings $p_{jq}$ are equal to the correlation between item *j* and factor *q*. Under the constraint of orthogonal factors, the cardinality-constrained problem defined in (5) can be solved efficiently in an alternating optimization scheme. Because (5) is not convex, this algorithm is also subject to local optima. Furthermore, the cardinality constraint itself is not a convex constraint; thus, the convergence of the AO procedure is not guaranteed (Huang et al., 2016).

### Stage 2: Estimating the structural model

In Stage 2, the factor loadings and factor scores obtained from Stage 1 can be used in several ways to study the structural relations among variables. The factor scores can directly be used with any suitable method for users’ purposes (e.g., multiple linear regression or path analysis); additional covariates can also be controlled for if desired. We present Algorithm 1 for solving the LASSO problem and Algorithm 2 for solving the cardinality-constrained problem in the special case of orthogonal factors in Appendix A and Appendix B, respectively.

### Model selection

The proposed estimation procedure for Stage 1 (exploratory approximate factor model) requires the following inputs: the number of factors *Q*, the number of nonzero loadings *C*, or the value of the tuning parameter $\lambda$. Proper values may be theory-based, but if such prior knowledge is lacking, a model selection strategy is needed. We propose a sequential strategy: first, the number of factors *Q* is determined based on either prior knowledge or (a combination) of various factor retention methods (e.g., parallel analysis (Horn, 1965), Kaiser–Guttman rule (Kaiser, 1960), scree test (Cattell, 1966), or a machine learning method (Goretzko & Bühner, 2020)); second, given *Q*, the algorithm searches through a sequence of candidate values for *K* or $\lambda$ and chooses the value for which the model yields the highest Index of Sparseness (IS). IS is an indicator developed to determine the level of sparseness in PCA (Gajjar et al., 2017; Trendafilov, 2014). In an extensive simulation study, Gu et al. (2019) showed that the IS resulted in the highest selection accuracy compared with other selection methods (i.e., BIC, cross-validation (CV), repeated double CV, Bolasso with CV, and stability selection). The formula for IS is as follows:

6 $$\begin{aligned} IS = PEV_{PCA} \times PEV_{rESEM} \times PS, \end{aligned}$$

where $PEV_{PCA}, PEV_{rESEM}$ and *PS* are the proportion of explained variance using ordinary PCA, the proportion of explained variance using Regularized ESEM, and the proportion of zero loadings, respectively. The IS value increases with increasing $PEV_{PCA}$, increasing $PEV_{rESEM}$, and the proportion of zero loadings. Thus, IS balances goodness-of-fit with the complexity of the solution (sparser solutions being less complex). A suitable range of *C* is *Q* (at least one nonzero loading per factor) to *JQ*. $\lambda$ ranges between 0 and $\lambda _{max}$ with $\lambda _{max}$ being the smallest value of $\lambda$ that makes $(J-1)$ elements become zero per factor.

### Related methods

The objectives of our two proposed methods are: (a) dealing with HDLSS settings, (b) deriving simple structure of the loading matrix with exact zero loadings to get interpretable factors, and (c) exploring relationships among the factors or with observed covariates/outcome variables. This section reviews existing methods related to ours. Their key features are summarized in Table 3. They can be categorized into three main groups: penalized maximum likelihood (ML) methods, (penalized )least-squares methods, and Bayesian methods.

The methods that use a penalized ML approach are RegSEM (Regularized SEM, Jacobucci, 2017), lslx (Penalized ML SEM Huang, 2020; Huang et al., 2017), and PSEM-Mplus (Penalized SEM in Mplus, Asparouhov & Muthén, 2024). They are all one-stage methods that add penalties to the ML discrepancy function with some fundamental differences. RegSEM and lslx both use similar types of shrinkage penalties to reduce the number of free parameters, stabilize estimation, and obtain interpretable solutions, with penalty parameters selected using information criteria or cross-validation. However, the two methods rely on different optimization algorithms, which results in different performance (Huang, 2020). In contrast, PSEM-Mplus primarily aims at estimating models that are unidentified under ordinary ML. For instance, to deal with rotational freedom in an EFA model, PSEM-Mplus can incorporate the Geomin rotation criterion as a penalty. When this penalty is lightly imposed, as recommended by Asparouhov and Muthén (2024, 2025), the resulting estimates will be highly similar to the rotation solution, and the log-likelihood fit is not sacrificed. Its penalty parameters are user-specified rather than tuned through data-driven methods. None of these penalized ML approaches were specifically proposed as solutions to high-dimensional data problems, and their performance has been evaluated only for complex models (i.e., models with many free parameters) in traditional low-dimensional settings.

Structured Factor Analysis (SFA, Cho & Hwang, 2023) is a two-stage least-squares method. In the first stage, SFA uses an alternating least-squares procedure to estimate the measurement parameters and factor scores. In the second stage, the estimated factor scores are used to estimate the structural parameters. SFA was proposed to avoid improper solutions (e.g., negative residual variances, non-positive-definite covariance matrices) that often occur in the traditional covariance-based SEM approach, and to derive a probability distribution for all feasible factor scores rather than obtaining a single unbiased estimate of factor scores. The latter makes it possible to draw probabilistic inferences about individuals’ true factor scores. Note that the method assumes a confirmatory measurement model rather than an exploratory one.

Regularized Generalized Structured Component Analysis (Regularized IGSCA, Cho et al., 2025) is a one-stage least-squares method, in which penalties (LASSO and Ridge) were added to the IGSCA objective function, separately for loadings and structural coefficients. IGSCA (Hwang et al., 2021) is a one-step framework that aims at simultaneously estimating common factors and components, allowing for hybrid models. The penalty is included to deal with multicollinearity problems (Ridge penalty) and perform variable selection (LASSO penalty). Regularized IGSCA uses K-fold cross-validation to determine the tuning parameter that controls the penalty strength. The method was demonstrated using the conventional SEM contexts only.

The Bayesian framework is an alternative approach for handling complex SEM models. Bayesian estimation, typically implemented via Markov chain Monte Carlo (MCMC), does not rely on large-sample asymptotics and can therefore provide stable estimates, valid uncertainty quantification, and improved performance in complex settings where the number of parameters is large relative to the sample size (Marcoulides et al., 2023) or high-dimensional settings through the use of shrinkage priors (van Erp, 2023). However, the MCMC procedures can be computationally intensive. Moreover, Bayesian estimation depends strongly on prior specification: diffuse default (noninformative) priors can lead to substantial bias in small samples (McNeish, 2016; Rosseel, 2020). Thus, both prior specification and model evaluation can be challenging and require careful judgment (Marcoulides, 2018; Muthén & Asparouhov, 2012; Van Erp et al., 2018). A full comparison of Bayesian SEM and frequentist regularization approaches is beyond the scope of this paper, and interested readers are referred to the relevant literature (Jacobucci & Grimm, 2018; van Erp, 2023).

In short, our proposed method relates to the above-mentioned approaches in two main ways: it adopts a two-stage least-squares framework and uses regularization to obtain simple structure in the loading matrix. However, important differences remain: our method applies a single type of penalty (LASSO) or a cardinality constraint only to the measurement model (unlike penalized ML approaches and Regularized IGSCA), and in doing so, sacrifices some of the fit to impose simple structure (to some degree) on the loading matrix. Furthermore, the model was developed such that solutions can be computed for very large data (in terms of the number of observed variables, sample size, or both), and it allows for a fully exploratory measurement model. The strength used to impose sparseness of the loadings is determined using a data-driven criterion (i.e., the Index of Sparseness).

## Simulation study

A simulation study was conducted to evaluate the performance of our proposed method compared with related methods. We excluded Bayesian SEM to maintain a focus on frequentist approaches, and SFA because it is not applicable in exploratory settings. The six methods RegSEM, lslx, PSEM-Mplus, Regularized IGSCA, and the proposed approaches (cardinality-constrained and LASSO) were examined based on the following performance measures: recovery rate of the simple structure for the measurement model; absolute bias and variance for the estimated regression weights in the structural model.

### Setup

**Fig. 1:**
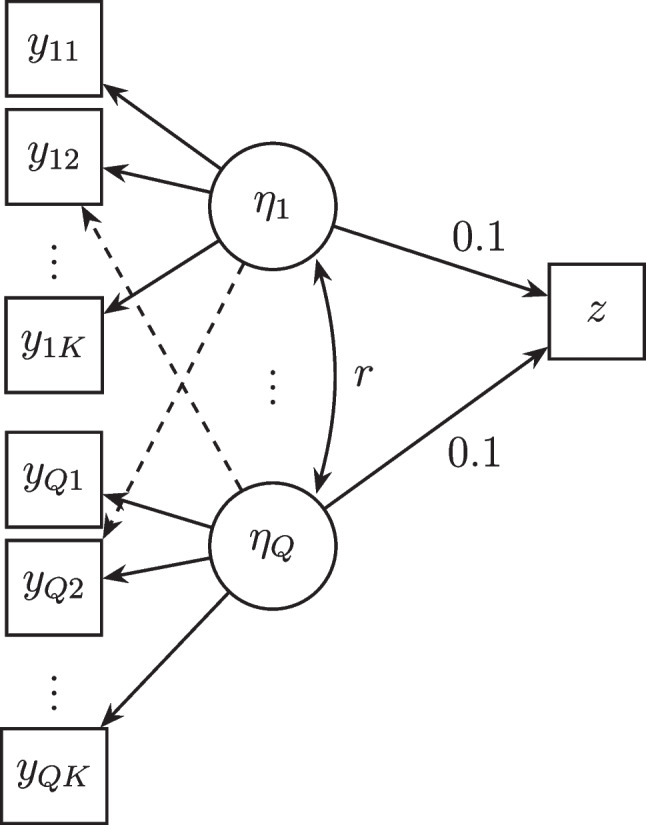
Population model to generate simulated data. The *dashed arrows* denote the cross-loadings

The simulated data were generated based on the population model in Fig. 1. For the traditional low-dimensional settings, we varied the following:

1. Sample size *N* at *3 levels*: 50, 100, 500.
2. Number of factors *Q* at *2 levels*: 3, 5.
3. Number of items *K* per factor at *2 levels*: 3, 5.
4. Cross-loadings at *2 levels*: 0, 0.5.
5. Item reliability (i.e., proportion of explained variance in each item by the factors) at *2 levels*: 0.3, 0.8.
6. Correlation *r* between factors *2 levels*: 0, 0.3.
7. Variance accounted for in the outcome $\textbf{z}$ by the factors (VAFz) at *2 levels*: 0.5, 0.9.

For the high-dimensional settings, we fixed $N = 50, Q = 5$ and varied the number of items *K* per factor at *3 levels*: 15, 30,  and 100 (which means in total there are 75, 150 and 500 items for a sample size of 50). The other criteria were varied in the same manner. The regression weights were fixed at 0.1 for all conditions. We set the primary loadings as $\textbf{P}_{primary} = \sqrt{0.6}$, and the cross-loadings $\textbf{P}_{cross} =.5$ which is a quite substantial cross-loading. The locations of the cross-loadings were specified in the same manner as in Rosseel and Loh (2022) and can be found in the detailed data generation procedure in Appendix D. All simulation design factors were crossed, and in each condition, 50 replicate data sets were generated, leading to 9600 low-dimensional datasets and 2400 high-dimensional datasets. In total, 10,400 datasets were generated and analyzed.

### Performance measures

The performance of all methods was compared using several measures that are defined as follows:

- The recovery of the simple structure with zero/nonzero recovery rate *PL*. It is the proportion of zero and nonzero true loadings $p_{jq}^{true}$ used to generate the data that is correctly recovered by the estimated loadings:
  7 $$\begin{aligned} PL \!=\! \frac{\# \,\text {of correctly nonzero loadings} + \#\,\text{of correctly identified zero loadings}}{\#\,\textrm {of loadings in }\textbf{P}_{true}}. \end{aligned}$$
- To assess the accuracy and precision of the estimated path coefficients in the structural model, we use the absolute bias and root mean squared error (RMSE).

**Fig. 2:**
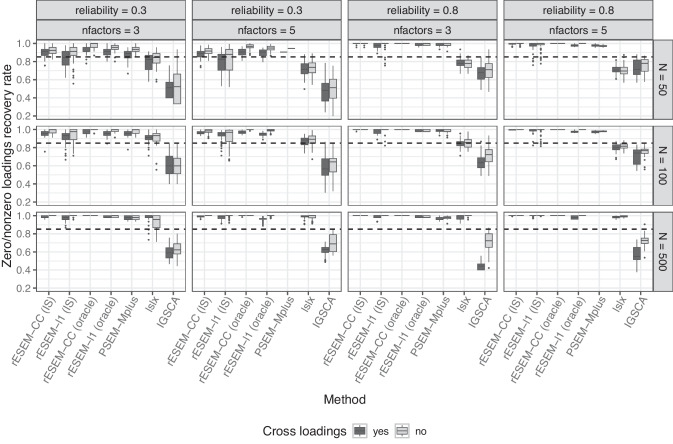
Percentage of correctly identified zero and nonzero loadings for orthogonal factors. *rESEM-l1* denotes the proposed Regularized ESEM using the LASSO penalty. *rESEM-CC* denotes the proposed Regularized ESEM using the cardinality-constrained approach; *(IS)* denotes the proposed method using the IS for model selection, while *(oracle)* uses the true number of (non)zero loadings. Improper and non-convergent results from *PSEM-Mplus* were excluded

### Analyses

The number of factors *Q* was treated as known in this simulation study. It is important to point out that deciding the number of factors in latent variable methods is an important task, yet not a straightforward one, which has raised extensive discussions. However, this is beyond the scope of this paper, and interested readers are referred to the literature dedicated to this matter (e.g., Auerswald & Moshagen, 2019; Goretzko, 2025). All other parameters (e.g., penalty tuning parameters, number of nonzero loadings, etc.) were treated as unknown for all methods.

**Fig. 3:**
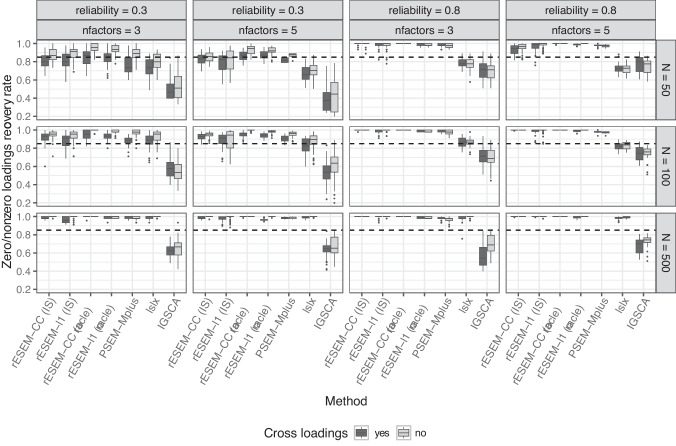
Percentage of correctly identified zero and nonzero loadings for correlated factors. *rESEM-l1* denotes the proposed Regularized ESEM using the LASSO penalty. *rESEM-CC* denotes the proposed Regularized ESEM using the cardinality-constrained approach; *(IS)* denotes the proposed method using the IS for model selection, while *(oracle)* uses the true number of (non)zero loadings. Improper and non-convergent results from *PSEM-Mplus* were excluded

For RegSEM, we chose the LASSO penalty in the algorithm and tuned the penalty parameter using cross-validation, following closely the authors’ tutorial paper (Li et al., 2021). We used lslx with the default setting with MCP penalty and tuned the (penalty and shape) parameters using the BIC. For PSEM-Mplus, the Geomin prior (as recommended in Asparouhov and Muthén (2024) for EFA/ESEM models) was used as the penalty function for all loadings: $p_{11} - p_{jq} \sim Geomin(Q, v)$, where *Q* is the number of factors and *v* takes the smallest value among (0.1, 1, 10, 100) for which the model converges. Since using the Geomin prior boils down to the same solution that would result from a rotation technique, the estimated factor loadings are not exactly zero. Thus, those that did not differ significantly from zero were considered to be zero in calculating the proportion of correctly recovered (non)zero loadings (significance level $\alpha =.05$). Lastly, Regularized IGSCA used the LASSO penalty with cross-validation to tune the penalty parameter. Both lslx and Regularized IGSCA failed to estimate the full model with measurement and structural models simultaneously; thus, we only used them to estimate the measurement model.

Our proposed methods require tuning the number of nonzero loadings (for the cardinality-constrained approach) and the penalty parameter (for the LASSO approach). To this end, the algorithm is run for a sequence of candidate values and chooses the one that maximizes the Index of Sparseness. We specified the range^2^ for the cardinality as *all* positive integers values between $[3\times Q, J \times Q]$, and 100 values of penalty parameters between $[N \times 0.1, \lambda _{max}]$, where $\lambda _{max}$ is the maximum penalty so that each factor has three items. We also used the proposed method with oracle information to disentangle the algorithmic performance of our method from the model selection performance. Here, the LASSO approach requires setting the value of the tuning parameter $\lambda$ to reach the correct number of (non)zero loadings. This was done using the binary search procedure where $\lambda$ is gradually adapted until the desired number of zero loadings is attained. That is, the model is estimated using a certain value of $\lambda$, which results in a number of zero loadings in the estimated loading matrix. Depending on whether the true loading matrix contains more or less zero loadings than the estimated one, $\lambda$ is increased or decreased, respectively. The cardinality-constrained approach is more straightforward: the number of (non)zeroloadings was given as direct input. Note that the factor solutions are subjected to permutational freedom and sign invariance. Thus, all possible permutations and sign configurations were considered, and the one yielding the highest Tucker’s congruence with the true factor loadings (Lorenzo-Seva & Ten Berge, 2006) was selected as the final solution.

**Fig. 4:**
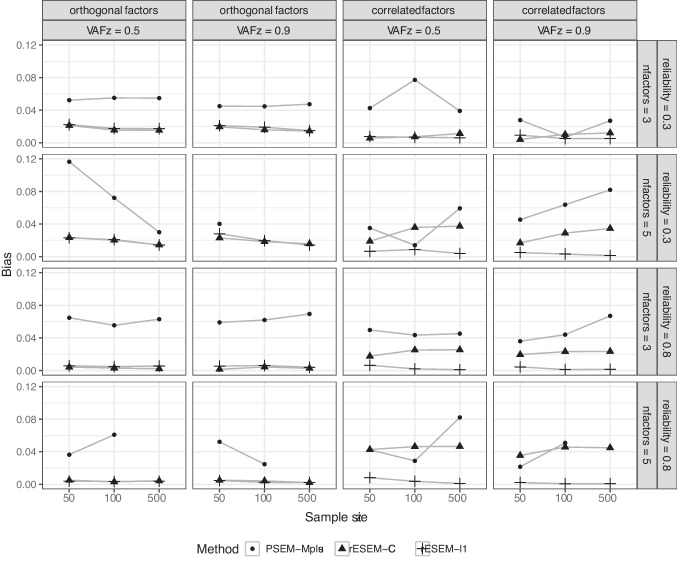
Average absolute bias of the estimated coefficients for the structural model. Results for *rESEM-l1* and *rESEM-CC* were based on model selection using the IS. Improper and non-convergent results from *PSEM-Mplus* were excluded

### Results

Interested readers can inspect interactively all simulation results with the R Shiny app at https://trale.shinyapps.io/rESEMextrasim/ (Le et al., 2025). It is worth noting that RegSEM struggled to converge and produce sensible results in even very simple settings (e.g., without cross-loadings, high reliability, etc.). Thus, we omitted RegSEM results from the simulation study.

#### Measurement model

We first discuss the results from the typical low-dimensional settings. Here, we report the extent to which each method recovered the underlying loadings structure. PSEM-Mplus and lslx both had non-convergence issues (i.e., no results were returned) in 4267 datasets and 37 datasets (out of 9600), respectively. Additionally, PSEM-Mplus produced improper results (i.e., negative residual variances) for 590 datasets. The proposed method (both the LASSO and cardinality-constrained approaches) converged in all datasets. Figures 2 and 3 show the zero/nonzero recovery rate of all methods across different conditions for orthogonal and correlated factors, respectively (for VAFz = 0.9 and five indicators per factor). As expected, the sample size, the number of factors, and item reliability affected all methods. That is, the recovery rate was higher for larger sample sizes and higher reliability, yet lower with a higher number of factors. The presence of cross-loadings proved to be challenging for all methods. Both of the proposed approaches, when using oracle information (i.e., the true number of (nonzero) loadings was used as input), had the highest rate of recovering the correct positions of zero and nonzero loadings. When the IS was used for model selection, the proposed cardinality-constrained approach still outperformed the other methods, followed by PSEM-Mplus and the proposed LASSO approach. However, it is important to note that the results for PSEM-Mplus were only based on 4743 datasets for which it converged with proper solutions, which is less than half of the total datasets. Regularized IGSCA had the lowest recovery rate.

**Fig. 5:**
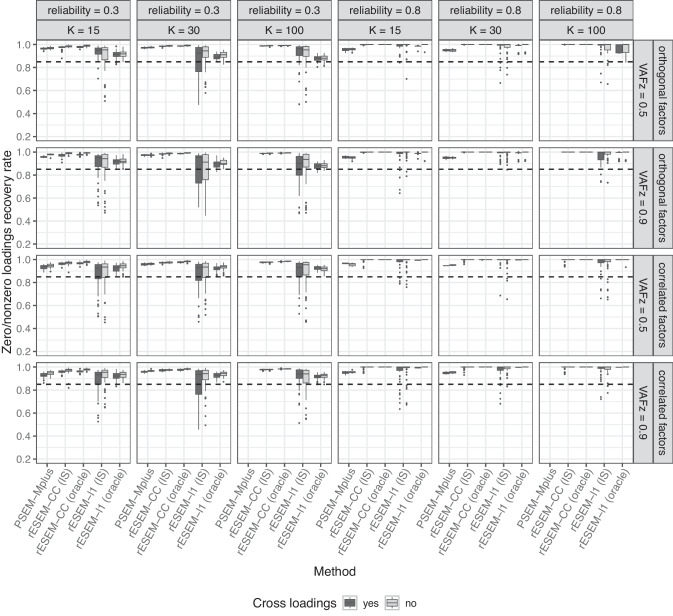
Percentage of correctly identified zero and nonzero loadings in high-dimensional settings. *rESEM-l1* denotes the proposed Regularized ESEM using the LASSO penalty. *rESEM-CC* denotes the proposed Regularized ESEM using the cardinality-constrained approach; *(IS)* denotes the proposed method using the IS for model selection, while *(oracle)* uses the true number of (non)zero loadings. Improper and non-convergent results from *PSEM-Mplus* were excluded

Focusing on the proposed Regularized ESEM, we saw an interesting pattern: the cardinality-constrained approach had slightly higher recovery rates than the LASSO approach, even when the factors were correlated in the data-generating model. Using Oracle information improved the recovery rate of the proposed method compared with the model selection procedure using the IS. The improvement is most noticeable for the proposed LASSO approach and in less ideal scenarios, namely, small sample sizes, low item reliability, and a larger number of factors. Furthermore, we recorded the percentage of datasets per condition for which the IS found the correct number of (non)zero loadings. The results are reported in Appendix E. It appeared that the accuracy of the IS was affected negatively by a larger number of factors and the presence of (moderate) cross-loadings. This pattern was stronger for the LASSO approach than the cardinality-constrained approach. Larger sample sizes seemed to improve the performance of the IS, especially in the case of the cardinality-constrained approach (i.e., the IS selected the correct cardinality for 100% of the datasets when $N = 500$). Note that this measure for the performance of the IS is a very strict criterion: any deviation between the true and selected number of (non)zero loadings, even by a single loading, is considered an incorrect selection. In high-dimensional settings (Table 6, the IS therefore appeared to perform worse because the larger number of loadings increases the chance of missing one. Both approaches in these analyses used a multi-start procedure, thus, it is of great interest to see how many different starts actually resulted in the same final solution. To this end, the loss value of each of 100 random starts was recorded and compared with the loss value of the final solution. The results are reported in Appendix F. Averaged across all conditions, the cardinality-constrained approach had a higher number of starts with the same loss value as the final one than the LASSO approach (i.e., 76 vs. 47).

**Fig. 6:**
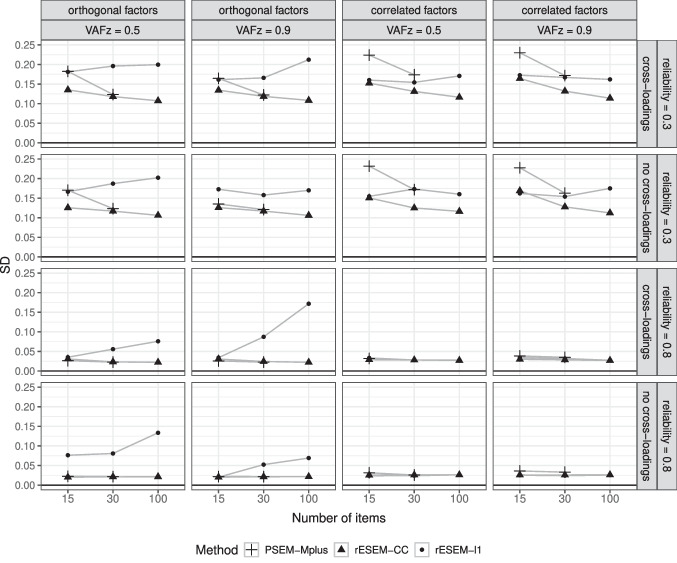
Average standard deviation of the estimated loading matrix across replications in the high-dimensional settings. *rESEM-l1* denotes the proposed Regularized ESEM using the LASSO penalty. *rESEM-CC* denotes the proposed Regularized ESEM using the cardinality-constrained approach. Improper and non-convergent results from *PSEM-Mplus* were excluded

#### Structural model

Figure 4 shows the absolute bias of the path coefficients across all conditions (for five items per factor and cross-loadings were present) in the low-dimensional settings. In the case of orthogonal factors, the two proposed approaches had almost indistinguishable bias. However, when the factors are correlated, the cardinality-constrained approach estimated the structural coefficients with much higher bias than the LASSO approach. This result is to be expected, given that the cardinality-constrained approach restricts the factors to be orthogonal. Thus, this approach is not safeguarded against local misspecification. Here, PSEM-Mplus appeared to be the most biased across almost all conditions. It is only less biased than the cardinality-constrained approach in a few scenarios with a high number of correlated factors and high item reliability. Again, note that more than half of PSEM-Mplus results were excluded due to non-convergence issues and improper solutions.

#### High-dimensional settings

In the high-dimensional settings, lslx and IGSCA failed to produce any results. The former method could not estimate any model because the sample covariance matrix is singular in high-dimensional settings. This prevented lslx from generating starting values for the estimated latent covariance matrix. PSEM-Mplus again failed to return any results in more than half of the datasets (i.e., 1528 out of 2400 datasets). Especially, this non-convergence issue occurred in all datasets with 500 observed variables.

The results for the measurement model by the proposed approaches and PSEM-Mplus in the high-dimensional settings are displayed in Fig. 5. Here, the same patterns as in the low-dimensional settings were observed: the performance of all methods was negatively affected by a lower reliability and the presence of cross-loadings; the cardinality-constrained outperformed the others in all conditions, and the LASSO approach was only better than the PSEM-Mplus when item reliability was high, and the oracle information was used. However, an important result to point out here is that while increasing the number of items per factor improved the recovery rates of all methods, PSEM-Mplus could not deal with situations with $K = 100$ (i.e., 500 items for a sample size of 50). Our proposed approaches did not encounter this problem. The stability of these estimated loadings in the high-dimensional settings was also evaluated by examining their (average) standard deviations (SD) across replications. The results in Fig. 6 suggested that the proposed cardinality-constrained approach had the lowest SD among the three methods. More importantly, the SD decreased as the number of items grew for both the proposed cardinality-constrained approach and PSEM-Mplus. This result confirms what has been established in the literature that studies the consistency of the approximate factor model in the high-dimensional setting (Daniele et al., 2025; Fan et al., 2021). However, this pattern did not seem to hold for the LASSO approach because the model selection procedure itself (using the IS to choose sparsity-inducing penalty) for the LASSO approach was already unstable (as seen in the previous sections), and thus, it could be exacerbated when there is a higher number of items.

The results for the structural model followed the same trend as the low-dimensional results, and thus, we do not report them here. Interested readers can inspect the detailed results using the Shiny app (Le et al., 2025). To summarize, PSEM-Mplus estimated the structural coefficients with the highest bias across almost all conditions. Both the proposed approaches were the least biased, but again, the proposed cardinality-constrained approach was substantially biased when factors were correlated in the data-generating model. The method, however, did have the lowest SD out of the three methods in all conditions.

### Summary

In short, the proposed method (both approaches) outperformed the other methods: the cardinality-constrained approach had the highest recovery rate of the zero/nonzero loadings regardless of the factors’ relationship, and the LASSO approach estimated the structural coefficients with the lowest bias (only compared with PSEM-Mplus). lslx and IGSCA failed to estimate complex models (i.e., models with both measurement and structural parts simultaneously) and also cannot deal with high-dimensional data. PSEM-Mplus and lslx both had non-convergence issues, with PSEM-Mplus having the worst convergence rate (i.e., it did not converge in more than 50% of the simulated datasets in both low- and high-dimensional settings).

It is important to point out that even though the cardinality-constrained approach outperformed the LASSO approach when estimating the measurement model for both orthogonal and correlated factors, this result did not hold for the structural part. That is, the cardinality-constrained approach produced much more biased structural coefficients than the LASSO approach when the factors were correlated in the population model. Thus, in an exploratory setting where no prior information about the factor correlations exists, it is advised to: first, use the cardinality-constrained approach to find the simple structure of the loadings; then, this simple structure can be used as a guide for the LASSO approach to tune the penalty parameter and estimate the factor scores.

## Empirical application

This section demonstrates the application of the two proposed methods using two empirical datasets: one is a traditional psychology questionnaire dataset while the other is an ultra-high-dimensional gene expression dataset (thousands of genes for just several dozen observations).

### Habitual stress recovery data and pre-processing

The first dataset concerns habitual strategies for stress recovery and their association with well-being and health in aging. Data was collected in 2023. In total, 421 individuals participated in the survey. For our analysis, we only included participants between 18 and 35 years as the “young” group and those 60 years old and older as the “old” age group. This results in a dataset with 415 observations. Data also contain missing values, which were imputed using multiple imputation with the ‘mice’ package in R (van Buuren & Groothuis-Oudshoorn, 2011). Here, we demonstrate how our proposed methods can be used to find the latent stress recovery strategies from self-reported questionnaire items and obtain factor scores for these strategies. The factor scores can then be used to explore how one’s recovery strategies are linked with their well-being.

**Table 1:** Loading matrix using the LASSO approach

Questionnaire	Items	External support	Cognitive reappraisal	Humor	Brooding	Suppression
IER	Co-distraction	0.771	0	0	0	0
	Co-suppression	0	0	0	0	0.874
	Co-brooding	0	0	0	0.656	0
	Physical affection	0.807	0	0	0	0
	Co-reappraisal	0.782	0	0	0	0
	Positive humor	0.513	0	0.635	0	0
	Negative humor	0	0	0.844	0	0
ERQ	Cognitive reappraisal	0	0.649	0	0	0
	Expressive suppression	0	0	0	0	0.874
COPE Brief	Active coping	0	0.776	0	0	0
	Planning	0	0.710	0	0	0
	Positive reframing	0	0.644	0	0	0
	Acceptance	0	0	0	-0.501	0
	Humor	0	0	0.802	0	0
	Religion	0	0	0	0	0
	Using emotional support	0.749	0	0	0	0
	Using instrumental support	0.737	0	0	0	0
	Self-distraction	0	0	0	0	0
	Denial	0	0	0	0.367	0
	Venting	0	0	0	0.405	0
	Substance	0	0	0	0	0
	Behavioral disengagement	0	0	0	0	0
	Self-blame	0	0	0	0.566	0
TCAQ	Thought control	0	0	0	-0.815	0
RSS	Reflection	0	0	0	0.663	0
	Brooding	0	0	0	0.844	0

#### Measures

Outcome variable The survey measured the participants’ satisfaction with life using a questionnaire of five items on a seven-point scale (Diener et al., 1985). We obtained a sum score of these five items to create a single outcome variable.

Exogenous factors The exogenous factors were the different latent stress recovery strategies. These strategies were measured using five different questionnaires: the COPE Brief (Carver, 1997) with 28 items on a four-point scale, Emotion regulation questionnaire (ERQ, Gross & John,^2003^) with ten items on a seven-point scale, Interpersonal Emotion Regulation in Close Relationship (IER, Horn, 2022) with 15 items on a five-point scale, Ruminative Responses Scale (RSS, Treynor et al., 2003) with ten items on a four-point scale, and Thought Control and Ability Questionnaire (TCAQ, Luciano et al., 2005) with 25 items on a five-point scale. For each questionnaire, we created sum scores for its subscales. In total, 26 sum scores were computed and used as observed variables for the measurement model. Variables were standardized prior to the analysis.

Control variables In this analysis, we controlled for participants’ education levels, gender, and age groups. Furthermore, we also examined whether the effects of stress recovery strategies differ between the young and old groups.

#### Results

Measurement model Using parallel analysis, we identified five exogenous factors from the five questionnaires measuring stress recovery strategies. Given this, we carried out the model selection procedure to determine the number of nonzero loadings. Ordinary PCA with five components accounted for 51.6% of the total variance. The maximum IS was achieved for a cardinality of 23 nonzero loadings using the cardinality-constrained approach (rESEM-CC), with five factors explaining 43.7% of the total variance (see Fig. 8 for the values of IS and proportion of explained variance as functions of the number of nonzero loadings). The penalty parameter of the LASSO approach (rESEM-l1) was tuned to reach the same number of nonzero loadings, which explained 44.6% of the total variance. Since we did not have prior knowledge, we chose to conduct the analysis using the LASSO approach to be able to explore the correlations of these five stress recovery strategies. The correlation matrix among the factors can be found in Appendix H. The final loading matrix is reported in Table 1.

Structural model The factor scores of the five stress recovery strategies were used in a regression analysis with Life Satisfaction as the outcome variable, controlling for participants’ gender, education level, age group, and the interactions between the stress recovery strategies and age group. As shown in Table 2, Brooding, Social Support, and Suppression strategies had significant effects on one’s life satisfaction level, controlling for the other predictors. Specifically, a higher level of using brooding and expressive suppression to deal with stress is associated with a lower level of one’s life satisfaction, controlling for other predictors. In contrast, individuals who tend to seek social support as a stress recovery strategy have a higher level of life satisfaction. Furthermore, the effect of using external support and expression suppression is stronger for the young group than the old group.

**Table 2:** Structural model parameters

Exogenous factors	rESEM-l1
(intercept)	0.214
External support	0.185**
Humor	0.091
Brooding	-0.416**
Cognitive reappraisal	0.037
Suppression	-0.169*
Female	-0.127
Other gender	-0.417
Young	-0.157
Lower vocational education	0.461
Vocational education	0.017
University of applied sciences	-0.155
Research university	-0.166
External support * Young	0.318*
Humor * Young	-0.149
Brooding * Young	-0.001
Cognitive reappraisal * Young	0.053
Suppression * Young	0.314*

*Note.* The outcome variable is Life Satisfaction

$$^{*}{p} < .05$$. $$^{**}{p} < .01$$

### The autism genetic data

To illustrate the proposed method as a summarization tool in high-dimensional settings, we use the gene expression data of lymphoblastoid cells to distinguish different types of autism (Nishimura et al., 2007). The dataset consists of 43,893 genetic markers measured for each of the 27 individuals, from which 14 are in the control group, six are affected with autism caused by a fragile X mutation (FMR1-FM), and seven are affected with autism caused by 15q11-q13 duplication (dup15q). All variables were standardized prior to the analyses. Three factors were chosen based on the original work of (Nishimura et al., 2007).

**Fig. 7:**
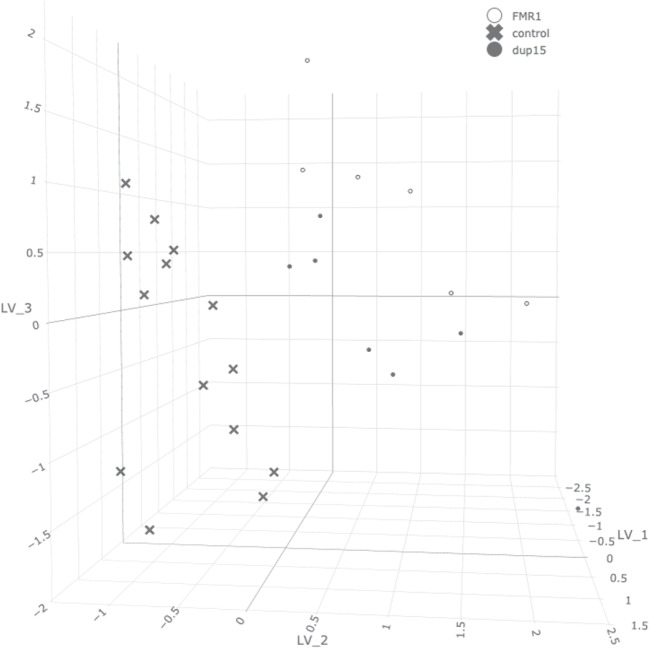
Scatterplot of the factor scores using cardinality constraint

**Fig. 8:**
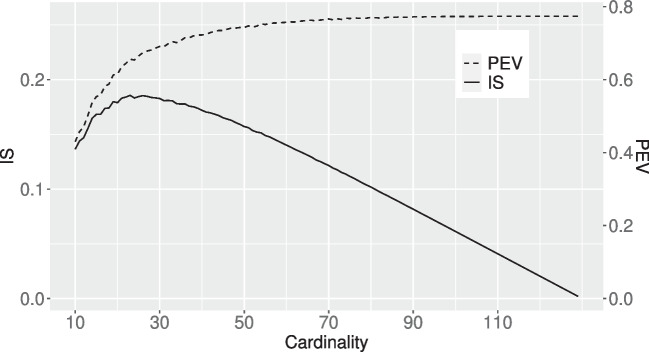
Index of sparseness (IS) and proportion of explained variance (PEV) against cardinality level

Ordinary PCA with three components explained 32% of the total variance. The maximum IS was achieved for the cardinality of 37,172 non-zero loadings for the cardinality-constrained approach, with the three factors explaining 25% of the total variance. Again, the LASSO approach was tuned with several trials and errors. Figure 7 presents the scatterplot of the factor scores^3^. As shown, the second factor clearly separates the control group from the two autism groups. This observation is in accordance with the result from Nishimura et al. (2007): the separation between the groups is the largest source of variation in the data. While the author used a data-driven approach (namely, maximizing the F statistic in an analysis of variance) to select a subset of 293 relevant variables which were subsequently used to construct genetic risk scores for autism, our method did not use such prior information and yet was still able to observe the distinction between groups.

## Discussion

In this paper, we propose a two-staged regularized exploratory SEM method. The most important contribution of this method lies in the first stage wherein we offer a unique exploratory approximate factor analysis that imposes simple structure on the loading matrix and can efficiently deal with high-dimensional data. The resulting measurement model can be used to indicate which variables load (i.e., have a non-zero loading) on each of the factors and followed by a *confirmatory* SEM/factor analysis. Additionally, factor scores are obtained in the second stage to directly investigate the structural relations among the variables in a manner appropriate to the users’ purposes. The method is rather easy to use, does converge, and comes with freely available code in R. It is important to note that the current paper does not propose a method to completely *replace* the traditional SEM method. Rather, our objectives are: Regularized ESEM can efficiently deal with exploratory and HDLSS settings, in which all other methods fail; (ii) Regularized ESEM produces a simple structure for the measurement model with *exact* zero loadings to ease interpretation, and (iii) Regularized ESEM is offered as a user-friendly algorithm in the free open-source statistical software R.

The proposed method uses the LASSO penalty to achieve a simple structure for the loading matrix, but we also pointed out the (more elegant) cardinality-constrained algorithm for the special case of orthogonal factors. Note that due to the different assumptions in factor relations (i.e., correlated versus orthogonal), the two approaches can lead to very different results. Especially, the cardinality-constrained approach is not robust against local misspecification: it produces highly biased estimates for the structural model when the factors are correlated in the population model. Thus, in an exploratory setting where no prior information is known, we recommend using the cardinality-constrained approach to determine the number of nonzero/zero loadings and their positions since its model selection procedure is more accurate and straightforward, regardless of the factor relations in the structural model. This can then be used as a guide for the LASSO approach to select the penalty parameter and estimate the structural paths.

There are several limitations to the current paper. Firstly, the paper did not propose a specific method for determining the number of factors. This is a nuanced task, especially in the high-dimensional settings where little prior knowledge is available. It often requires inspecting different results from different methods of extracting the number of factors, instead of using one single method (Auerswald & Moshagen, 2019). Although factor retention was not addressed in this paper, it is an important direction for future work. For instance, a more detailed exploration of this issue in the context of regularized methods and high-dimensional settings would provide more insights and completeness for the proposed framework. Secondly, there are three sources of uncertainty carried from the first to the second stage of the proposed method: (i) model selection uncertainty; (ii) parameter estimation uncertainty in Stage 1 (i.e., factor scores are based on loadings, which are themselves estimates); and (iii) factor scores are not free of measurement errors in the low-dimensional settings. A potential solution would be to draw inferences for Stage 2 using a type of parametric bootstrap method: data is sampled from a multivariate normal distribution using the correlation matrix of the original data. For each bootstrap sample, model selection is performed, and factor loadings and scores are estimated, which are then used to estimate the structural model. However, this remedy can be computationally intensive, and in the low-dimensional settings, it does not correct for the bias of the estimates caused by measurement errors. This issue of measurement error yielding biased estimates for the structural parameters in the low-dimensional setting might be resolved by accounting for unique factors (e.g., as done in IGSCA Cho et al., 2025; Hwang et al., 2021) or using some bias-correction methods (for an overview of step-wise methods in latent variable modeling, see Vermunt, 2024). Alternatively, one can use the nonzero/zero loadings structure obtained from the cardinality-constrained approach as prior information for a (semi)confirmatory method, such as in lavaan (Rosseel, 2012), Mplus (Muthén & Muthén, 1998-2017), lslx (Huang, 2020), and others, which would help reduce the number of parameters needed to be freely estimated in an exploratory model that often causes non-convergence issues for these methods (even in the low-dimensional case). This type of strategy is also known as EFA-based CFA (EFCA) in the literature and has been shown to estimate parameters with good accuracy and model fit (Nájera et al., 2023).

Apart from the aforementioned limitations, our work can be extended in several directions. For example, another topic of interest is the use of a cardinality constraint in more general settings. As seen in this paper, the cardinality constraint offers several advantages yet requires orthogonality of the factor scores. An algorithm that does not require this orthogonality constraint can be developed based on the numerical solution proposed by Adachi and Kiers (2017) in the regression context and Guerra-Urzola et al. (2023) in the sparse PCA context. Note that such an algorithm does not guarantee convergence and optimality, similarly to the proposed Regularized ESEM in this paper. A further topic of research is to examine the predictive power of the proposed method, which might be useful for applied researchers who are not only interested in explaining the underlying mechanisms but also in predicting certain outcomes. Another natural extension of the current method is to account for multi-group analyses, for which the latent factors can be compared across different groups of individuals.

## Figures and Tables

**Algorithm 1:**
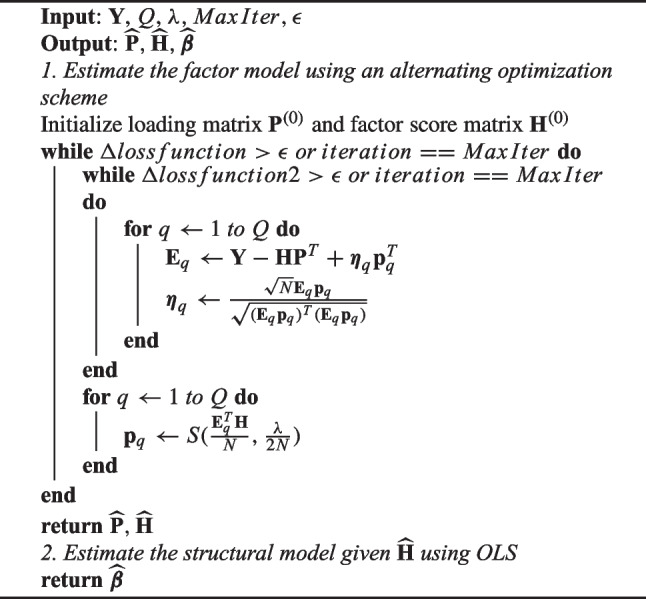
Regularized ESEM with the LASSO penalty (rESEM-l1)

**Algorithm 2:**
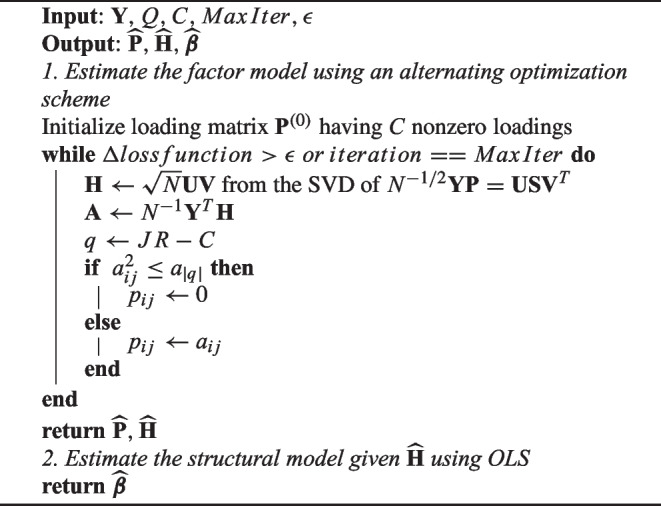
Regularized ESEM with cardinality constraint (rESEM-CC)

**Fig. 9:**
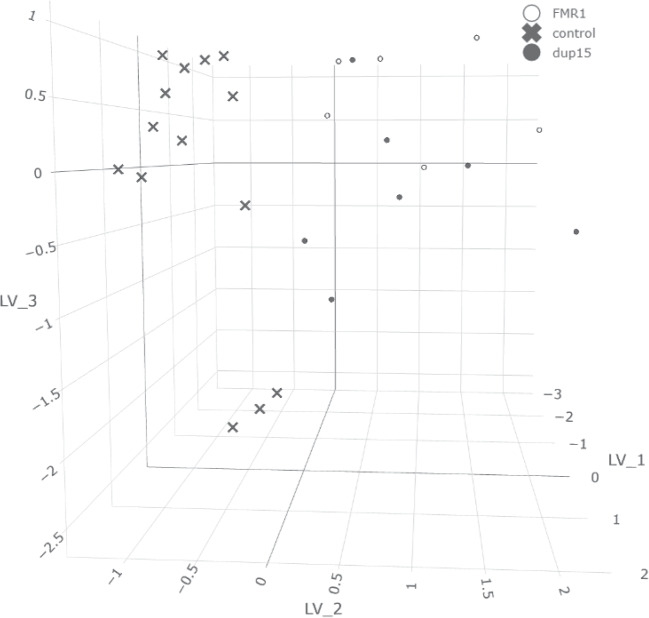
Three-dimensional scatterplot of the correlated factor scores obtained from the LASSO approach rESEM-l1

## Data Availability

The synthetic data and results reported in this manuscript can be generated using the code publicly available at https://github.com/trale97/regularizedESEM. This manuscript made use of two empirical data sets. The Habitual Stress Recovery Strategies data are not publicly available but can be made accessible from the author (Dr. Nicola Ballhausen at n.m.ballhausen@tilburguniversity.edu) upon reasonable request. The Autism Genetic data (Nishimura et al., 2007) is available at https://www.ncbi.nlm.nih.gov/geo/query/acc.cgi?acc=GSE7329.

## Appendix A Algorithm: Regularized ESEM with the LASSO penalty

Here, we present the mathematical derivations and detailed description of the algorithm for the method using the LASSO penalty.

1. Update of the factor scores conditional upon fixed values for the loadings is based on the following objective:

8 $$\begin{aligned} \min _{\eta _{11},\ldots ,\eta _{iq},\ldots ,\eta _{NQ}}&\sum _{j=1}^J\sum _{i=1}^N\left( y_{ij}-\sum _q\eta _{iq} p_{jq}\right) ^2 + \lambda \sum _{j=1}^J\sum _{q=1}^Q|p_{jq}|_1 \nonumber \\ \text {subject to }&\sum _i\eta _{iq}^2=N \quad \forall q=1,...Q. \end{aligned}$$

Equation (8) can be rewritten in matrix form as follows:

9 $$\begin{aligned} \min _{\boldsymbol{\eta }_1,\ldots ,\boldsymbol{\eta }_q,\ldots ,\boldsymbol{\eta }_Q}&||\textbf{Y} - \sum _q \boldsymbol{\eta }_q \textbf{p}_q^T||^2 + \sum _q\lambda |\textbf{p}_q|_1 \nonumber \\ \text {subject to }&\boldsymbol{\eta }_q^T\boldsymbol{\eta }_q = N \quad \forall q=1,...,Q. \end{aligned}$$

Using the method of Lagrange Multipliers, we obtain the Lagrangian function

10 $$\begin{aligned} \mathcal {L}&= ||\textbf{Y}-\sum _q \boldsymbol{\eta }_q \textbf{p}_q^T||^2 - \mu (\boldsymbol{\eta }_q^T \boldsymbol{\eta }_q-N)\nonumber \\&= ||\textbf{Y}-\sum _{t \ne q}\boldsymbol{\eta }_t\textbf{p}_t^T - \boldsymbol{\eta }_q\textbf{p}_q^T||^2 - \mu (\boldsymbol{\eta }_q^T \boldsymbol{\eta }_q-N)\nonumber \\&= ||\textbf{E}_q - \boldsymbol{\eta }_q\textbf{p}_q^T||^2 - \mu (\boldsymbol{\eta }_q^T \boldsymbol{\eta }_q - N) \end{aligned}$$

Solving $\frac{\partial \mathcal {L}}{\partial \boldsymbol{\eta }_q} = 0$ and $\frac{\partial \mathcal {L}}{\partial \mu } = 0$, we have $\boldsymbol{\eta }_q = \frac{\sqrt{N}\textbf{E}_q\textbf{p}_q}{\sqrt{(\textbf{E}_q\textbf{p}_q)^T(\textbf{E}_q\textbf{p}_q)}}$.

2. To update the loading matrix $\textbf{P}$ a coordinate descent procedure is used. First note that the objective function is separable in the variables:

11 $$\begin{aligned} \min _{p_{11},\ldots ,p_{pq},\ldots ,p_{JQ}}&\sum _{j=1}^J\sum _{i=1}^N\left( y_{ij}-\sum _q\eta _{iq} p_{jq}\right) ^2 + \lambda \sum _{j=1}^J\sum _{q=1}^Q|p_{jq}|_1\nonumber \\&= \sum _{j=1}^J\left( \sum _{i=1}^N\left( y_{ij}-\sum _q\eta _{iq} p_{jq}\right) ^2 \right) + \lambda \sum _{j=1}^J\sum _{q=1}^Q|p_{jq}|_1, \end{aligned}$$

implying that we can optimize per variable *j*, this is solving for each *j*,

$$\begin{aligned}&\min _{p_{j1},\ldots ,p_{jq},\ldots ,p_{jQ}}\sum _i(y_{ij}-\sum _q\eta _{iq} p_{jq})^2 + \sum _q\lambda |p_{jq}|. \end{aligned}$$

Coordinate descent relies on a conditional optimization scheme were each of the $p_{jq}$ is estimated $\forall q=1,\ldots ,Q$ in turn and using an iterative scheme. With some rewriting, the objective for obtaining the conditional estimate of $p_{jq}$ becomes

$$\begin{aligned}&\min _{p_{jq}}\sum _i(y_{ij}-\sum _{t \ne q}\eta _{it}p_{jt} - \eta _{iq}p_{jq})^2 + \sum _q\lambda |p_{jq}|\\&= \sum _i(e_{ij}^r - \eta _{iq}p_{jq})^2 + \sum _{t \ne q}\lambda |p_{jt}| + \lambda |p_{jq}| \end{aligned}$$

which is minimized by

$$\begin{aligned}&p_{jq} = \frac{2\sum _ie_{ij}\eta _{iq} - \lambda }{2\sum _i\eta _{iq}^2} = \frac{2\sum _ie_{ij}\eta _{iq} - \lambda }{2N} \text { if } \sum _ie_{ij}\eta _{iq} > \frac{\lambda }{2}\\&p_{jq} = \frac{2\sum _ie_{ij}\eta _{iq} + \lambda }{2\sum _it_{iq}^2} = \frac{2\sum _ie_{ij}\eta _{iq} + \lambda }{2N} \text { if } \sum _ie_{ij}\eta _{iq} < -\frac{\lambda }{2}\\&p_{jq} = 0 \text { if }|\sum _ie_{ij}\eta _{iq}|\le \frac{\lambda }{2}. \end{aligned}$$

The solution corresponds to the univariate soft thresholding operator derived in the context of the univariate LASSO regression problem (Friedman et al., 2010).

Using the estimated factor score to obtain the path coefficients in the structural part with OLS (or other methods suitable for users’ purposes), we present the algorithm for LSLV-LASSO:

## Appendix B Algorithm: Regularized ESEM with cardinality constraint

With orthogonal factors, finding the best subset of loadings to (5) is not an NP-hard problem but can be efficiently solved in an alternating optimization scheme in which the optimization criterion is decomposed into two parts:

12 $$\begin{aligned} \sum _{j=1}^J\sum _{i=1}^N\left( y_{ij} \!-\! \sum _{q=1}^Q\eta _{iq} p_{jq}\right) ^2&\!\!=\!\! \sum _{j=1}^J\sum _{i=1}^N\left( y_{ij} \!-\! \sum _{q=1}^Q\eta _{iq} a_{jq}\right) ^2\nonumber \\&+ N\left( \sum _{j=1}^J\sum _{q=1}^Q a_{jq} \!-\! p_{jq}\right) ^2. \end{aligned}$$

with $a_{jq} = \frac{1}{N}\sum _iy_{ij}\eta _{iq}$. The decomposition in  (12) holds true if $\sum _i\eta _{iq}^2 = N \quad \forall q=1,...,Q$ and $\sum _i\eta _{iq}\eta _{it} \quad \forall q \ne t$.

The first term does not involve the loadings $p_{jq}$, while the second can be easily minimized under the cardinality constraint by keeping *C* loadings with the highest absolute value and setting the remaining loadings to zero. The main complexity of the procedure only involves sorting the loading matrix $\textbf{P}$ of size $J \times Q$, and thus can scale up to a large number of variables. Even though this method has previously been introduced as unpenalized sparse principal component analysis, we would like to point out that, because of the cardinality constraint, the component scores cannot be written as a linear combination of the variables (unlike with PCA). Hence, here we have a sparse exploratory factor analysis method with reflective indicators: the loadings express the strength of the association of the score vectors with the observed variables.

Equation (12) can be rewritten in matrix form as follows:

13 $$\begin{aligned} ||\textbf{Y} - \textbf{HP}^T||^2 = ||\textbf{Y} - \textbf{HA}^T||^2 + N||\textbf{A}-\textbf{P}||^2, \end{aligned}$$

with $\textbf{A} = \frac{1}{N}\textbf{Y}^T\textbf{H}$ ($\textbf{H}$ is the factor score matrix). Then, $\textbf{H}$ is attained by minimizing the expanded loss function

14 $$\begin{aligned} \text {tr}\textbf{Y}^T\textbf{Y} \!+\! \text {tr}\textbf{P}\textbf{H}^T\textbf{H}\textbf{P}^T \!-\! 2\text {tr}\textbf{Y}^T\textbf{HP} \!=\! N\text {tr}\textbf{S}+N\text {tr}\textbf{PP}^T \!-\! 2N\text {tr}\textbf{AP}^T, \end{aligned}$$

with $\textbf{S} = N^{-1}\textbf{Y}^T\textbf{X}$. Hence, we have

15 $$\begin{aligned} \textbf{H} = \sqrt{N}\textbf{UV}^T, \end{aligned}$$

with $\textbf{U}$ and $\textbf{V}$ from the SVD of $\frac{1}{\sqrt{N}}\textbf{YP} = \textbf{USV}^T$.

For a given $\textbf{H}$, minimizing the loss function (13) over constrained $\textbf{P}$ is equivalent to minimizing:

16 $$\begin{aligned} ||\textbf{A}-\textbf{P}||^2 = \sum _{(j,q) \in Z}a_{jq}^2 + \sum _{(j,q) \in Z^*}(a_{jq} - p_{jq})^2 \ge \sum _{(j,q) \in Z}a_{jq}^2, \end{aligned}$$

with *Z* being the set of $k = J \times Q - C$ indexes (*j*, *q*) for $p_{jq} = 0$ and $Z^*$ being the set of indexes for $p_{jq} \ne 0$. Thus, (16) is minimized for $\textbf{P} = (p_{jq})$ being

17 $$\begin{aligned} p_{jq}= {\left\{ \begin{array}{ll} 0, & \text {if}\ a_{jq}^2 \le a_{|k|}^2 \\ a_{jq}, & \text {otherwise} \end{array}\right. } \end{aligned}$$

Using the estimated factor scores to obtain the path coefficients in the structural part with OLS (or other methods suitable for users’ purposes), we present the algorithm for CCLSLV:

## Appendix C Summary of related methods

**Table 3:** Summary of related methods

Method	Reference	Type	Penalty	Software
RegSEM	Jacobucci et al. (2016)	Penalized ML (one-stage)	$l_1,l_2$, elastic net	R package
lslx	Huang et al. (2017); Huang (2020)	Penalized ML (one-stage)	$l_1,l_2$, elastic net, SCAD, MCP (default)	R package
PSEM-Mplus	Asparouhov and Muthén (2024)	Penalized ML (one-stage)	Prior*	Mplus
SFA	Cho and Hwang (2023)	LS (two-stage)	No	MATLAB
Regularized IGSCA	Cho et al. (2025)	Penalized LS (one-stage)	$l_1,l_2$	MATLAB
Bayesian SEM	Bayesian (one-stage)	Shrinkage priors (e.g., normal, Laplace, horseshoe, spike-and-slab, etc.)	Various	

*Note*. ML = Maximum-likelihood; LS = Least-squares; l1 = LASSO; l2 = Ridge; SCAD = smoothly clipped absolute deviation penalty; MCP = minimax concave penalty

## Appendix D Data generation for simulation study

The setup and data generation procedure for the simulation study was inspired by Bollen et al. (2024); Rosseel and Loh (2022), which consists of the following steps:

1. Generate loadings $\textbf{P}_{J \times Q}$: The primary loadings were fixed as $\sqrt{.6}$ (similarly done by De Roover and Vermunt (2019); The cross-loadings were fixed as 0.5 and their locations were generated as follows: for each factor $\boldsymbol{\eta }_q$, take the middle item from the block of items that load primarily on this factor and assign this item a cross-loading of 0.5 for the next factor $\boldsymbol{\eta }_{q+1}$.
2. Generate factor scores $\textbf{H}_{N\times Q}$ from a multivariate normal distribution $MVN(\textbf{0}, \boldsymbol{\Phi })$, where $\boldsymbol{\Phi }$ is the correlation matrix between the factors. In the case of orthogonal factors, $\boldsymbol{\Phi } = \textbf{I}_{Q\times Q}$.
3. Generate the residuals $\textbf{E}_{N \times J}$ from a multivariate normal distribution $MVN(\textbf{0}, \boldsymbol{\Theta })$ where the diagonal of $\boldsymbol{\Theta }$ are the unique variance of the items. The diagonal of $\boldsymbol{\Theta }$ was generated to match the desired level of item reliability.
4. Generate the observed variables $\textbf{Y} = \textbf{HP}^T + \textbf{E}$.
5. Generate the observed outcome variable $\textbf{z} = \textbf{H}\boldsymbol{\beta } + \textbf{E}_z$, where each $\beta$ is fixed as 0.1 and $\textbf{E}_z$ was generated to match the desired VAFz (variance accounted for in $\textbf{z}$ by the factors) level.

## Appendix E Accuracy of Index of Sparseness as a model selection criterion

**Table 4:** Percentage of datasets per condition for which IS selected the correct number of (non)zero loadings (for orthogonal factors and VAFz = 0.9)

Items per factor	Number of factors	Sample size	Cross-loadings	rESEM-CC	rESEM-l1
3	3	50	yes	78	24
5	3	50	yes	76	24
3	5	50	yes	73	20
5	5	50	yes	70	30
3	3	100	yes	88	30
5	3	100	yes	88	32
3	5	100	yes	80	34
5	5	100	yes	77	38
3	3	500	yes	100	49
5	3	500	yes	100	55
3	5	500	yes	100	48
5	5	500	yes	100	64
3	3	50	no	79	54
5	3	50	no	70	48
3	5	50	no	71	39
5	5	50	no	65	38
3	3	100	no	98	74
5	3	100	no	98	64
3	5	100	no	98	60
5	5	100	no	96	50
3	3	500	no	100	96
5	3	500	no	100	90
3	5	500	no	100	92
5	5	500	no	100	96

*Note.* The results for VAFz = 0.5 had a similar trend. Reported values are rounded to whole numbers

**Table 5:** Percentage of datasets per condition for which IS selected the correct number of (non)zero loadings (for correlated factors and VAFz = 0.9)

Items per factor	Number of factors	Sample size	Cross-loadings	rESEM-CC	rESEM-l1
3	3	50	yes	80	30
5	3	50	yes	64	30
3	5	50	yes	68	24
5	5	50	yes	62	20
3	3	100	yes	88	34
5	3	100	yes	80	38
3	5	100	yes	72	40
5	5	100	yes	75	36
3	3	500	yes	100	54
5	3	500	yes	100	59
3	5	500	yes	91	50
5	5	500	yes	96	62
3	3	50	no	62	52
5	3	50	no	61	44
3	5	50	no	70	34
5	5	50	no	57	34
3	3	100	no	86	64
5	3	100	no	85	55
3	5	100	no	85	51
5	5	100	no	72	50
3	3	500	no	100	96
5	3	500	no	100	90
3	5	500	no	100	88
5	5	500	no	100	84

*Note.* The results for VAFz = 0.5 had a similar trend

**Table 6:** Percentage of datasets per condition for which IS selected the correct number of (non)zero loadings (high-dimensional scenario)

Items per factor	Reliability	Factors correlation	Cross-loadings	rESEM-CC	rESEM-l1
15	0.3	0	yes	50	1
30	0.3	0	yes	31	2
100	0.3	0	yes	15	0
15	0.8	0	yes	69	38
30	0.8	0	yes	92	38
100	0.8	0	yes	97	28
15	0.3	0.3	yes	31	1
30	0.3	0.3	yes	19	1
100	0.3	0.3	yes	2	2
15	0.8	0.3	yes	83	41
30	0.8	0.3	yes	80	34
100	0.8	0.3	yes	80	29
15	0.3	0	no	50	3
30	0.3	0	no	47	0
100	0.3	0	no	9	0
15	0.8	0	no	100	78
30	0.8	0	no	100	65
100	0.8	0	no	100	62
15	0.3	0.3	no	19	1
30	0.3	0.3	no	6	2
100	0.3	0.3	no	0	1
15	0.8	0.3	no	100	69
30	0.8	0.3	no	100	68
100	0.8	0.3	no	100	53

## Appendix F Number of starts resulting in the same final solution by the proposed Regularized ESEM

**Table 7:** Number of starts resulting in the same final solution by Regularized ESEM out of 100 random starts

Number of factors	Sample	Factors correlation	cross-loadings	rESEM-CC	rESEM-l1
3	50	0	yes	76	48
5	50	0	yes	62	46
3	100	0	yes	84	47
5	100	0	yes	74	46
3	500	0	yes	93	47
5	500	0	yes	92	46
3	50	0.3	yes	72	48
5	50	0.3	yes	58	47
3	100	0.3	yes	80	48
5	100	0.3	yes	62	47
3	500	0.3	yes	94	49
5	500	0.3	yes	82	46
3	50	0	no	79	47
5	50	0	no	59	46
3	100	0	no	87	47
5	100	0	no	68	46
3	500	0	no	92	47
5	500	0	no	79	45
3	50	0.3	no	74	47
5	50	0.3	no	53	46
3	100	0.3	no	82	48
5	100	0.3	no	58	46
3	500	0.3	no	92	48
5	500	0.3	no	74	46
Total				76	47

*Note.* The results were averaged across 50 replications and some conditions

## Appendix H Factors correlation obtained by the LASSO penalty approach rESEM-l1

**Table 8:** Correlation matrix of the five factor scores obtained by the Lasso penalty approach

	External	Suppression	Cognitive	Humor	Brooding
	support		reappraisal		
External support	1.000	-0.434	0.233	-0.021	0.185
Suppression	-0.434	1.000	-0.119	-0.241	0.162
Cognitive reappraisal	0.233	-0.119	1.000	0.020	-0.151
Humor	-0.021	-0.241	0.020	1.000	-0.179
Brooding	0.185	0.162	-0.151	-0.179	1.000

## Footnotes

1 In exceptional cases, uniqueness is not guaranteed, e.g., in case of highly correlating factors.

2 This range ensures the minimum cardinality is three items per factor and the maximum cardinality is all loadings are nonzero

3 The scatterplots resulting from the two methods are very similar, hence, we only reported the one using cardinality constraint. The other plot using the LASSO penalty can be found in Appendix I

## Code availability

The implementation of the proposed method was done in R and can be found, along with the code to reproduce the results in this manuscript, at https://github.com/trale97/regularizedESEM.

## Declarations

### Open Practices Statements

The manuscript made use of simulated data and two empirical datasets. The code for data reproduction and analyses is available at https://github.com/trale97/regularizedESEM. One of the empirical datasets is not publicly available but can be made accessible from the author (Dr. Nicola Ballhausen at n.m.ballhausen@tilburguniversity.edu) upon reasonable request. The code for its analysis is also available at the same GitHub repository.
